# Necroptosis-related diseases are regulated by the unfolded protein response during endoplasmic reticulum stress

**DOI:** 10.1016/j.gendis.2026.102060

**Published:** 2026-01-30

**Authors:** Tong Li, Tian Su, Sha Tang, Kun Cao, Pengbing Hua, Jingbiao Gao, Hongwang Cui, Tongmeng Jiang

**Affiliations:** a NHC Key Laboratory of Tropical Disease Control, Engineering Research Center for Hainan Bio-Smart Materials and Bio-Medical Devices, Key Laboratory of Hainan Functional Materials and Molecular Imaging, School of Life Sciences and Medical Technology, Hainan Medical University, Haikou, Hainan 571199, China; b Key Laboratory of Emergency and Trauma of Ministry of Education, Department of Emergency Surgery, Key Laboratory of Hainan Trauma and Disaster Rescue, The First Affiliated Hospital, Hainan Medical University, Haikou, Hainan 570102, China; c Key Laboratory of Emergency and Trauma of Ministry of Education, Key Laboratory of Haikou Trauma, Key Laboratory of Hainan Trauma and Disaster Rescue, The First Affiliated Hospital, Hainan Medical University, Haikou, Hainan 571199, China

**Keywords:** ATF6, Endoplasmic reticulum (ER) stress, IRE1α, Necroptosis, PERK, Unfolded protein response (UPR)

## Abstract

In recent years, research has highlighted that necroptosis plays a significant role in various diseases, suggesting that studying the pathways of necroptosis may offer novel avenues for developing treatments for these conditions. The classical necroptosis pathway involves the activation and phosphorylation of RIPK1, RIPK3, and MLKL. Moreover, the unfolded protein response (UPR) during endoplasmic reticulum (ER) stress is crucial in regulating necroptosis. ER stress impacts cell survival by activating the UPR, which subsequently influences downstream PERK, IRE1α, and ATF6 pathways. This paper will elucidate the signaling mechanisms within the classical necroptosis pathway and the three UPR pathways involved in ER stress. Additionally, this review will focus on the functional mechanisms of the UPR pathways in necroptosis and the latest research advancements on how these pathways regulate necroptosis and influence the progression of related diseases.

## Introduction

Diseases associated with necroptosis primarily refer to those in which the regulatory mechanisms involve cell necroptosis during their pathogenesis. Necroptosis is a tightly regulated form of cell death that differs from apoptosis; it often involves the rupture of the cell membrane and the release of cellular contents, which subsequently triggers inflammatory responses ^1^. Recent studies have shown that necroptosis plays a crucial role in various diseases, including musculoskeletal disorders ^2^, cardiovascular diseases ^3^, digestive diseases ^4^, renal diseases ^5^, and tumors ^6^. Researchers have investigated how targeting necroptosis pathways can lead to new therapeutic approaches for these conditions ^6^. For instance, inhibiting key molecules such as receptor-interacting serine/threonine kinase 1 (RIPK1), RIPK3, or mixed lineage kinase domain-like protein (MLKL) might effectively prevent or slow down necroptosis, thereby reducing inflammation and tissue damage ^6^. A deeper understanding of the regulatory mechanisms of necroptosis could provide new strategies and targets for treating these diseases.

The endoplasmic reticulum (ER) is the primary site for protein synthesis, folding, and quality control within cells. ER stress (ERS) occurs when there is a disturbance in protein folding, leading to the activation of the unfolded protein response (UPR) to restore protein stability ^7^. If UPR persists, it may result in necroptosis ^8^. ERS can regulate necroptosis by increasing UPR and activating the PKR-like ER-resident kinase (PERK), inositol-requiring enzyme 1 alpha (IRE1α), and activating transcription factor 6 (ATF6) pathways ^7^. Necroptosis, a form of cell death independent of caspases (cysteine-aspartic proteases), is triggered under specific pathological stimuli. RIPK1 and RIPK3 are key kinases in the necroptosis pathway, executing cell death through the phosphorylation of MLKL ^9^. ERS may contribute to the pathogenesis of various diseases, including neurodegenerative diseases, cardiovascular diseases, metabolic disorders, and cancers ^10^, by modulating necroptosis. Understanding these molecular mechanisms is crucial and may aid in the discovery of novel therapies.

In this review, we delineate the intricate signaling cascades underpinning the canonical necroptosis pathway, as well as the three distinct UPR pathways that come into play during ERS. Furthermore, we explore the role these pivotal processes play in the pathogenesis of musculoskeletal, cardiovascular, gastrointestinal, renal disorders, and neoplasms. Elucidating the mechanisms by which ERS governs necroptosis and keeping abreast of the latest scientific advancements may pave the way for novel preventive, diagnostic, and therapeutic approaches, thus providing fresh avenues for the management of these associated maladies.

## Necroptosis and related pathway

### Necroptosis

Research on regulated cell death primarily focuses on mechanisms such as necroptosis, cuproptosis, apoptosis, pyroptosis, and ferroptosis ^11^. Additionally, other mechanisms of cell death are gradually becoming focal points of academic interest. These include ERS-induced apoptosis ^12^, oxidative stress-mediated cell death ^13^, cell death triggered by aerobic glycolysis inhibition ^14^, aponecrosis ^15^, parthanatos ^16^, lysosome-mediated cell death ^17^, and NETosis ^18^. Necroptosis is a form of cell death independent of Caspase-8 (CASP8), involving key molecules such as tumor necrosis factor-α (TNF-α), RIPK1, RIPK3, and MLKL ^7^. This mode of cell death is distinct from apoptosis and autophagy, manifesting as a lytic form of death characterized by the release of intracellular contents, including damage-associated molecular patterns (DAMPs). The release of DAMP molecules can trigger the activation of immune responses ^9^. Necroptosis is a finely regulated cell death mechanism, marked by oxidative stress, random DNA degradation, and the release of pro-inflammatory cytokines during the process of cell death. The necrosome, a hallmark structure, plays a crucial role in this type of cell death ^19^. Morphologically, necroptosis is characterized by significant changes in cell morphology, including rounding of the cell, swelling of the cytoplasm, and expansion of organelles, with no chromatin condensation. Ultimately, the rupture of the cell membrane leads to the release of cellular contents, resulting in cell death. Under electron microscopy, cells undergoing necroptosis exhibit lysosomal rupture, deformation of other organelles, and partial alterations in the nuclear ultrastructure, such as chromatin dissolution ^20^.

### TNF-α/RIPK1/RIPK3/MLKL pathway

The TNF-α/RIPK1/RIPK3/MLKL pathway is the most classic pathway involved in the occurrence of necroptosis, activated by TNF-α binding to its receptor ^21^. Within the cell, this pathway involves several key molecules, including RIPK1, RIPK3, and MLKL ^22^. The interaction between TNF-α and tumor necrosis factor receptor 1 (TNFR1) is crucial. When TNF-α binds to TNFR1, it triggers the formation of complex I, which comprises tumor necrosis factor receptor type 1-associated death domain protein (TRADD), RIPK1, tumor necrosis factor receptor-associated factor 2/5 (TRAF2/5), cellular inhibitor of apoptosis 1/2 (cIAP1/2), and linear ubiquitin chain assembly complex (LUBAC) ^20^.

Complex I plays a critical role in determining cell fate, with its ubiquitination status guiding different signaling pathways under various conditions. The formation of complex I depends on the ubiquitination of RIPK1 mediated by cIAP, which subsequently recruits the inhibitor of nuclear factor-kappa B kinase (IKK) complex and activates the nuclear factor kappa-B (NF-κB) pathway, leading to the release of interferons and the initiation of an inflammatory response ^23^. Conversely, when TNF-α stimulation leads to the formation of complex Ia by TRADD with FADD and CASP8, it results in apoptosis. If the deubiquitination of RIPK1 promotes the formation of complex IIb with FADD, RIPK1/3, and CASP8, then necroptosis occurs ^24^. In this context, the inhibition of CASP8 is crucial for the formation of the necrosome. Upon inhibition of CASP8, activated RIPK1 associates with RIPK3, leading to the activation of RIPK3. Subsequently, RIPK3 undergoes phosphorylation and activates MLKL. Once activated, MLKL oligomerizes, and its 4HB domain^25^ permeabilizes the cell membrane, triggering necroptosis ^9^.

## UPR regulation of necroptosis in ERS

### UPR

ERS is a cellular response to the accumulation of misfolded or unfolded proteins within the ER lumen and disturbances in calcium ion balance. This stress state activates a series of signaling pathways, including the UPR, the ER overload response, and the caspase-12-mediated apoptosis pathway, all aimed at restoring ER homeostasis ^26^. The ER is the primary site within the cell responsible for protein synthesis, folding, transport, calcium homeostasis regulation, and lipid biosynthesis. When the ER's function is compromised by internal or external stimuli, such as oxidative stress or disruptions in calcium homeostasis, it leads to the accumulation of misfolded or unfolded proteins within the ER, triggering the UPR ^7^. More importantly, UPR also participates in physiological processes such as cartilage development ^27^ and pathological disorders, including osteoporosis ^28^.

**Figure 1:**
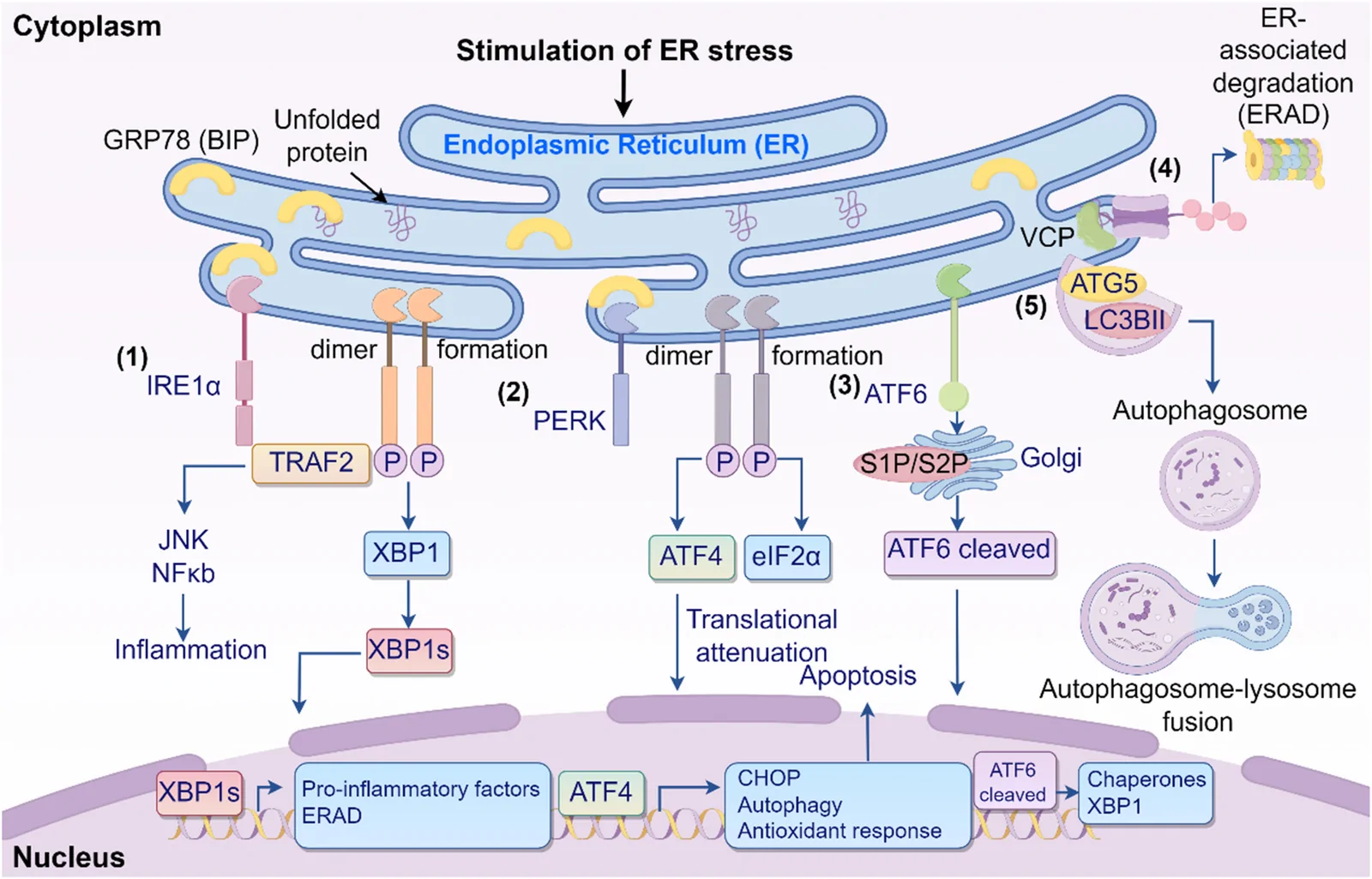
Molecular mechanism of unfolded protein response in endoplasmic reticulum (ER) stress. When ER stress occurs, the transmembrane sensors PERK, IRE1α, and ATF6 dissociate from GRP78, resulting in the activation of downstream pathways, including i) IRE1α-mediated inflammation, ii) PERK-mediated apoptosis, iii) ATF6-mediated inflammation, iv) VCP-mediated ERAD, and v) ATG5-mediated autophagosome-lysosome fusion process. Among them, PERK, IRE1α, and ATF6 pathways have been demonstrated to be related to necroptosis. The figure was created by Figdraw.

The UPR involves three major ER transmembrane protein sensors: IRE1α, PERK, and ATF6. Under normal conditions, these sensors are bound to the immunoglobulin heavy chain binding protein (GRP78/BIP). During ERS, these transmembrane sensors dissociate from BIP, resulting in the activation of downstream pathways, including IRE1α-mediated inflammation, PERK-mediated apoptosis, ATF6-mediated inflammation, VCP-mediated ERAD, and ATG5-mediated autophagosome-lysosome fusion process ^29^, as illustrated in Figure 1. A moderate UPR response promotes cell survival, whereas excessive UPR activation may lead to necroptosis ^30^. In particular, PERK, IRE1α, and ATF6 pathways have been demonstrated to be related to necroptosis and are discussed in the following sections.

### PERK pathway

The PERK pathway plays a crucial role in regulating protein synthesis, folding, oxidative stress, and other aspects during ERS. PERK is an ERS sensor protein that is ubiquitously present in eukaryotic cells ^26^. Unfolded proteins bind to PERK, inducing a conformational change that promotes PERK oligomerization and autophosphorylation. Once PERK is activated, the eukaryotic initiation factor 2-alpha (eIF2α) undergoes phosphorylation, rendering it inactive. This reduces protein synthesis activity and decreases the production of new proteins, thereby helping to alleviate the protein load within the cell ^31^.

If the ERS state persists, the translation of ATF4 mRNA, a downstream protein of eIF2α, is activated. This, in turn, promotes the activation of the CCAAT/enhancer-binding protein homologous protein (CHOP) promoter, which controls the expression of target genes ^32^. ATF4 and CHOP are key transcription factors downstream of the PERK/eIF2α pathway. When ERS cannot be resolved, the forced expression of ATF4 and CHOP increases protein synthesis ^33^. ATF4 is an upstream regulator of CHOP and can up-regulate CHOP expression. CHOP is a pro-apoptotic protein that can induce apoptosis through various mechanisms. Therefore, when the unfolded protein response persists during ERS, continuous activation of the PERK pathway can lead to a series of downstream events such as ATP depletion, oxidative stress, and cell death ^33^^,^^34^.

Previous research has primarily focused on the core mechanisms of the PERK pathway downstream of the UPR during ERS. However, there is relatively little research on the role of the PERK signaling pathway within the upstream and downstream gene regulatory networks and its association with related diseases, particularly concerning the pathway's involvement in necroptosis. Recent studies have shown that the PERK pathway can regulate necroptosis in various diseases. Zhylkibayev et al ^35^ found in their study on nitrogen mustard-induced ocular toxicity in mice that nitrogen mustard could lead to decreased corneal function, which may be associated with the activation of UPR and PERK pathways, as well as necroptosis. This suggests a potential link between the PERK pathway's regulation of necroptosis and corneal diseases. Even the mechanism by which ERS regulates necroptosis through the PERK pathway within the UPR is well-established. However, the specific mechanisms of this regulation in different cell types require further investigation and will be discussed in the following sections.

### IRE1α pathway

The IRE1α pathway is the most conserved pathway within the UPR and is crucial for maintaining ERS and protein stability. As illustrated in Figure 1, IRE1α is a transmembrane serine/threonine (Ser/Thr) protein kinase and endoribonuclease that resides in the endoplasmic reticulum. It is a single transmembrane protein with both kinase and ribonuclease activity functions. During ERS, IRE1α transitions from dimerization to oligomerization, which triggers the phosphorylation of positive regulatory sites within the kinase domain^36^. Once the ribonuclease function of IRE1α is activated, it removes a 26-nucleotide intron from the mRNA of the UPR-specific transcription factor X-box binding protein 1 (XBP1). This splicing converts the unspliced form, XBP1u, into the spliced form, XBP1s, thereby enhancing the protein processing capacity of the endoplasmic reticulum. XBP1s may increase the transcriptional translocation of several UPR and ER-associated degradation (ERAD) related genes into the nucleus, thereby alleviating ERS ^37^.

Previous studies have primarily focused on the association between the IRE1α pathway downstream of the UPR during endoplasmic ERS and cellular apoptosis and senescence. However, research on the role of the IRE1α pathway in necroptosis is relatively limited. Liang et al ^29^ found that activation of the IRE1α pathway could regulate XBP1 splicing in necroptotic cells, ultimately triggering an inflammatory response. Therefore, the IRE1α pathway may promote necroptosis by activating inflammatory responses ^38^^,^^39^. However, research by Qu et al ^40^ demonstrated that in a hepatocyte ischemia-reperfusion injury model, activation of macrophage RIPK3 could regulate inflammation responses caused by specific genes via the IRE1α pathway. This suggests that in immune cells, necroptosis might trigger immune responses, thereby inducing ERS. Additionally, findings by Li and colleagues ^38^ indicate that activation of the IRE1 pathway can up-regulate the expression of TNF-α and various inflammatory factors, thereby triggering RIPK1/RIPK3/MLKL pathway-dependent necroptosis. This study provides direct evidence that ERS-induced activation of the IRE1α pathway downstream of the UPR can regulate TNF-α/RIPK1/RIPK3/MLKL pathway-mediated necroptosis.

Overall, the IRE1α pathway primarily influences cell survival *in vivo* by activating inflammation. If ERS regulates necroptosis through this pathway, upon activation of the IRE1α pathway, it can indirectly affect the activity or expression of RIPK1 and RIPK3 by modulating intracellular inflammatory responses and signaling pathways. This includes the activation of the IRE1α and c-Jun N-terminal kinase (JNK) pathway, leading to necroptosis ^41^.

### ATF6 pathway

As depicted in Figure 1, the ATF6 pathway primarily regulates functions related to protein synthesis, secretion, and inflammation. ATF6 is an ER membrane protein with two subtypes: ATF6α and ATF6β. ATF6α plays a major role in transcriptional activation and cellular protection, while ATF6β may act as an endogenous inhibitor regulating ATF6α signaling. The N-terminal of these proteins has transcriptional activation functions, and the C-terminal contains multiple BIP binding sites and Golgi localization signals ^42^.

During ERS, the activation of ATF6 involves the reduction of protein disulfide bonds and translocation from the ER to the Golgi apparatus; its active form is rapidly degraded following ERS activation ^43^. In the Golgi, specific proteases such as site-1 protease (S1P) and S2P cleave ATF6, releasing its extracellular domain into the cytoplasm. The released fragments, now possessing transcriptional activation activity, can enter the nucleus and bind to specific promoter regions, initiating the expression of a series of ERS response-related genes to help the cell alleviate ER stress and restore normal ER function ^43^.

ATF6β can also be activated to some extent during ERS, translocating to the Golgi for cleavage, producing transcriptionally active fragments that enter the nucleus ^44^. Although its gene expression profile overlaps partially with that of ATF6α, it shows a lower degree of activation for certain genes ^44^. In some cases, ATF6α and ATF6β may work synergistically to regulate the expression of genes related to the ERS response. Previous research on the role of the ATF6 pathway downstream of the UPR in ERS has predominantly focused on its involvement in apoptosis and autophagy. Numerous studies have confirmed that the ATF6 pathway can promote both apoptosis and autophagy ^45^. However, recent studies have demonstrated that the ATF6 pathway also plays a role in necroptosis. For instance, research by Li et al ^46^ found that ERS could activate oxidative stress and trigger NF-κB-mediated necroptosis, indicating that ERS can regulate necroptosis by promoting oxidative stress. Another study observed that in mouse distal colon cells, up-regulation of ATF6 expression was associated with increased levels of RIPK1/3, suggesting that activation of the ATF6 pathway within the UPR can lead to necroptosis ^47^. Conversely, Chen et al ^48^ discovered that during liver injury, ERS could activate ATF6 to up-regulate hepatic expression of alpha-fetoprotein, which in turn mitigates ERS, thereby reducing hepatocyte necroptosis. Interestingly, baicalein has been shown to alleviate necroptosis via the ATF6 pathway ^41^.

In summary, while the ATF6 pathway can indeed regulate necroptosis, certain drug interventions and the influence of specific proteins *in vivo* can attenuate this process. This suggests that ATF6-mediated necroptosis triggered by UPR might be modulated under specific conditions. In instances of sustained ERS, this pathway may play a role in regulating necroptosis ^44^. The ATF6 pathway may indirectly influence the activity or expression of RIPK1 and RIPK3 by modulating intracellular inflammatory responses and signaling pathways. As the terminal effector protein in the necroptosis pathway, MLKL can undergo conformational changes and insert into the cell membrane after being phosphorylated and activated by RIPK3, compromising membrane integrity and leading to cell necrosis. In ERS-related necroptosis, the ATF6 pathway might influence MLKL activation and function indirectly through upstream signal transmission, thereby promoting necroptosis ^49^.

## The role of ERS in regulating necroptosis-related diseases

### Orthopedic and dental diseases

Necroptosis plays a significant role in bone and cartilage development as well as musculoskeletal and dental diseases. Researchers have discovered that the RIPK1/RIPK3/MLKL pathway-mediated necroptosis is one of the primary modes of cell death in osteocytes in postmenopausal osteoporosis ^20^. Our group has further validated that TNF-α facilitates osteocyte necroptosis in postmenopausal osteoporosis by inducing the up-regulation of TLR4 ^50^. ERS may also influence multiple musculoskeletal processes. Reports suggest that the UPR in ERS is associated with orthopedic diseases such as osteoporosis ^28^, osteoarthritis ^51^, chondrogenesis ^27^, osteogenesis imperfecta ^52^, lumbar degenerative disease ^53^, and osteosarcoma ^54^. In the osteoarthritis, osteoporosis, and osteosarcoma conditions, the RIPK1/RIPK3/MLKL-mediated necroptosis plays distinct roles: RIPK1/RIPK3/MLKL mediates necroptosis in cartilage cells driving disease progression, while RIPK3/MLKL triggers osteoclast cell death associated with bone loss. Additionally, both UPR/ERS pathways are activated, which are linked to cartilage homeostasis disruption, bone instability with osteopenia, inflammatory responses, survival mechanisms, and tumor biology.

The three pathways of the UPR are crucial in regulating the formation and death of osteoblasts. Excessive ERS can induce osteoblast apoptosis by activating downstream UPR pathways, leading to osteoporosis ^55^. However, it remains unclear whether UPR regulates necroptosis in osteoblasts and osteocytes or affects osteoclast function. Research by Guo et al ^26^ confirmed that the PERK pathway in UPR indeed regulated osteoclasts and found that ERS activation in osteoclasts could enhance autophagy. Whether ERS participates in osteoclast necroptosis still needs verification. If a link is found, it could provide a theoretical basis for new therapeutic strategies to maintain bone homeostasis and prevent bone loss in osteoporosis patients. Furthermore, Zhao et al ^56^ found that TNF-α could enhance oxidative stress in bone marrow mesenchymal stem cells, inducing ERS, and inhibiting ERS can mitigate TNF-α-induced inflammation. As mentioned earlier, the RIPK1/RIPK3/MLKL signaling pathway mechanism in necroptosis highlights TNF-α as a key upstream protein in this process. Thus, inhibiting ERS might also reduce osteoblast necroptosis mediated by the RIPK1/RIPK3/MLKL pathway.

In the hyperplastic tissue surrounding implants in free fibula flaps, stress-induced cellular changes were observed, with immunohistochemistry showing up-regulation of UPR markers such as PERK, XBP1, NFκB, and MLKL, with PERK expression significantly increased. These findings suggest that the formation of granulation tissue after oral surgery may be associated with the PERK pathway's regulation of necroptosis ^57^.

**Figure 2:**
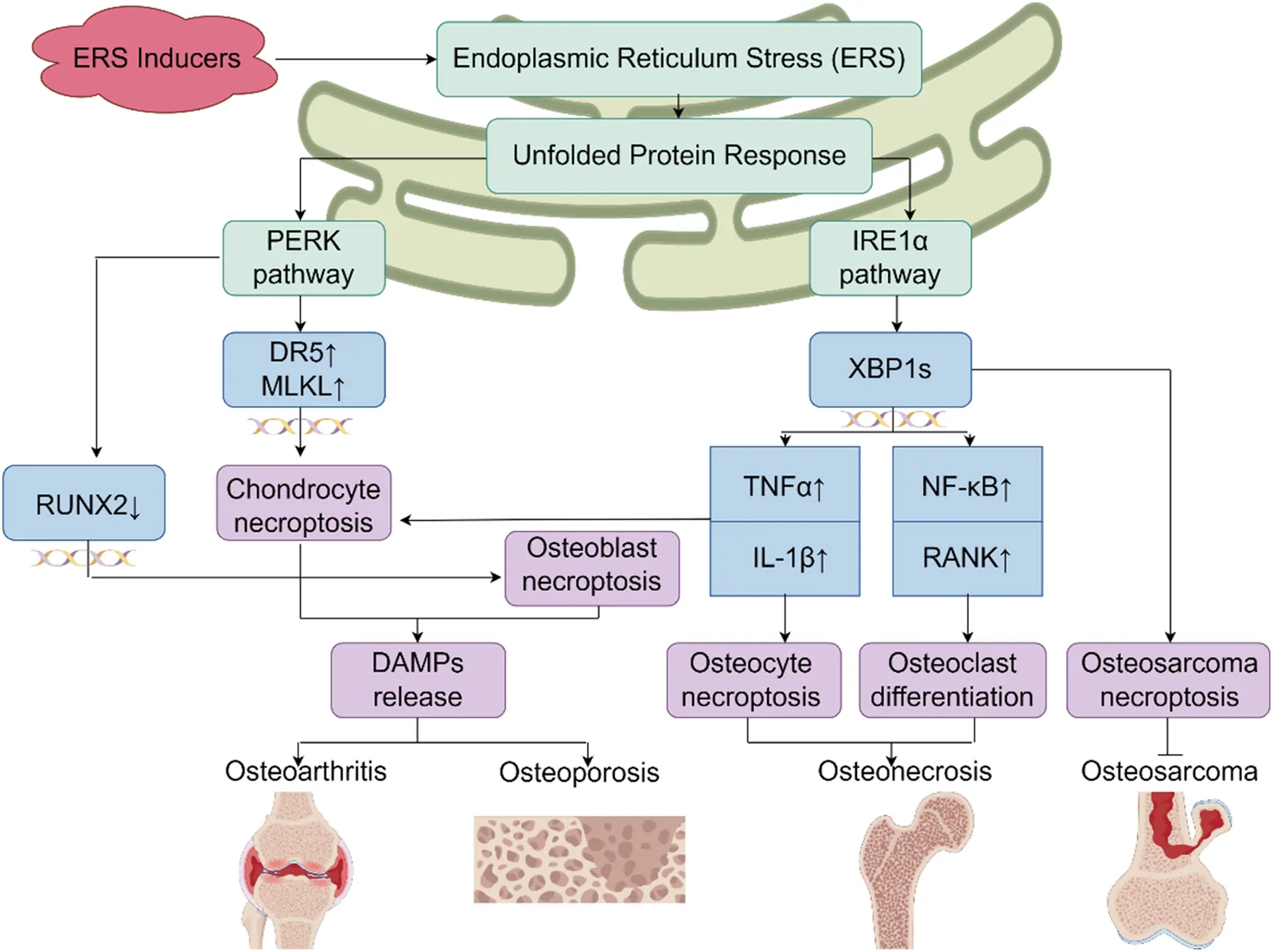
Endoplasmic reticulum stress (ERS) and unfolded protein response (UPR) regulate necroptosis in the musculoskeletal system. ERS inducers trigger UPR pathways, including PERK and IRE1α in chondrocyte, osteoblast, osteoclast, and osteosarcoma cell necroptosis to accelerate osteoarthritis, osteoporosis, and osteonecrosis, while inhibiting osteosarcoma. The role of ATF6-mediated ERS in necroptosis during musculoskeletal disease pathogenesis remains unclear. The figure was created by Figdraw.

In conclusion, we propose that the ERS processes may activate RIPK1/RIPK3/MLKL by up-regulating the inflammatory cytokine TNF-α, leading to necroptosis in osteoblasts, osteoclasts, osteosarcoma cells, and chondrocytes. This process may contribute to the development of osteoarthritis, osteoporosis, and bone necrosis and inhibit the progression of osteosarcoma (Fig. 2). Future research confirming the specific roles of downstream UPR signaling pathways in osteoblasts and osteoclasts could provide a theoretical foundation for treating osteoporosis, osteoarthritis, osteochondrosis, osteogenesis imperfecta, and osteosarcoma.

### Cardiovascular diseases

Recent studies have highlighted the critical role of necroptosis in maintaining myocardial homeostasis, ischemic injury, pathological remodeling, and heart failure, providing strong supporting evidence, in particular, the RIPK3/MLKL-dependent necroptosis activated by UPR/ERS ^58^. In cardiac diseases such as myocardial infarction and heart failure, the activation of ERS is closely linked to the necroptosis of cardiomyocytes. Following myocardial infarction, the activation of ERS leads to the initiation of the UPR, affecting the survival and function of cardiomyocytes ^59^. Although our current understanding of the regulatory role of UPR in ERS-mediated necroptosis within cardiovascular diseases is limited, there is evidence indicating that the PERK pathway of ERS plays a crucial upstream regulatory role in the necroptosis of vascular smooth muscle cells and further accelerates atherosclerosis ^60^. In a mouse model of cigarette tar-induced atherosclerosis, Bai et al ^60^ discovered that tar can activate the PERK pathway, which in turn activates downstream signaling pathways, leading to calcium ion metabolism imbalance and reactive oxygen species production, ultimately resulting in RIPK3-dependent necroptosis. The use of the ERS inhibitor 4-phenylbutyric acid (4-PBA) significantly reduced plaque progression and necroptosis of vascular smooth muscle cells ^61^. This indicates that the PERK pathway in ERS can regulate necroptosis and suggests that the RIPK1/RIPK3/MLKL signaling pathway might be downstream of the PERK pathway. Furthermore, research has confirmed the significant role of UPR-regulated necroptosis in ethanol-induced dilated cardiomyopathy ^62^^,^^63^. For instance, studies using a necroptosis model in H9c2 cardiomyocytes have shown up-regulation of GRP78, CHOP, and CASP12 proteins ^62^, while the ERS inhibitor 4-PBA was able to mitigate myocardial damage induced by necroptosis, thereby alleviating cardiomyopathy. Glycogen synthase kinase-3β (GSK-3β) may be a downstream protein of CHOP and might participate in promoting vascular endothelial cell damage ^64^. This evidence suggests that in cardiovascular diseases, ERS can regulate necroptosis through the UPR pathway.

**Figure 3:**
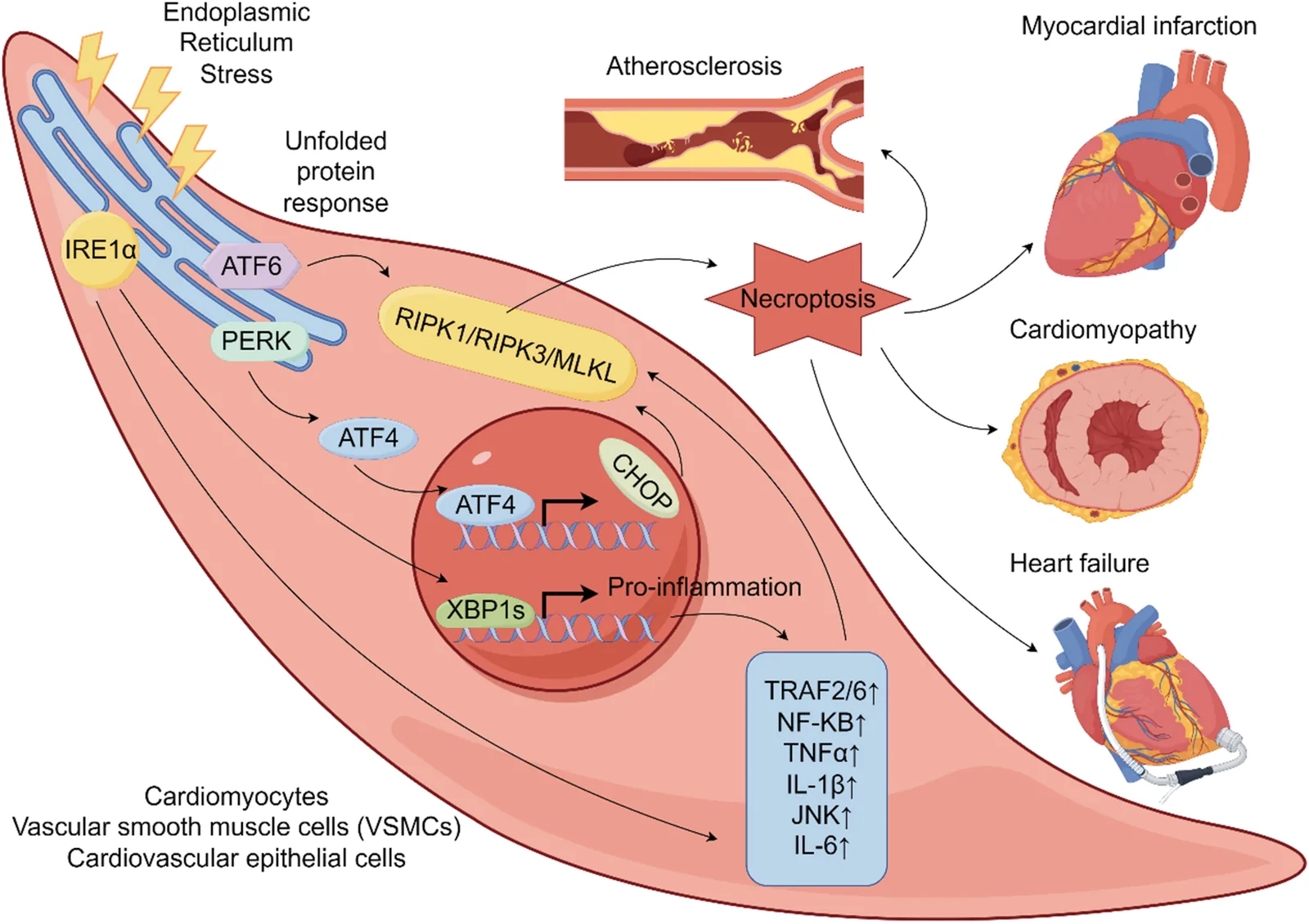
Endoplasmic reticulum stress can regulate necroptosis in cardiovascular diseases through the downstream PERK, IRE1α, and ATF6 pathways by targeting RIPK1/RIPK3/MLKL signaling. The figure was created by Figdraw.

In conclusion, we propose that ERS may induce necroptosis in cardiomyocytes, vascular smooth muscle cells and cardiovascular epithelial cells by activating RIPK1/RIPK3/MLKL-dependent necroptosis through up-regulation of inflammatory factors such as TNF-α, IL-6, IL-12, and IL-1β, thereby promoting disease progression (Fig. 3). While the roles of other pathways remain unclear, these findings imply that UPR-mediated regulation of necroptosis in ERS might become a novel therapeutic target for cardiovascular diseases. The precise mechanisms of ERS in cardiovascular diseases require further research for clarification. Future studies need to delve deeper into the role of ERS in various cardiovascular diseases involving cell death and explore therapeutic strategies targeting ERS pathways, aiming to provide new directions for the treatment of cardiovascular diseases.

### Digestive diseases

As previously mentioned, scientists have revealed that all three pathways of the UPR in ERS play critical regulatory roles in hepatocyte necroptosis ^48^. Notably, the PERK pathway may also have a regulatory role in necroptosis in liver cancer cells, but its effects can be quite distinct. For example, in a study by Zein et al ^65^, combined cisplatin–sunitinib treatment in liver cancer cells led to a reduction in pRIPK3/RIPK3 expression, while PERK and CASP8 expression increased significantly. This suggests that in liver cancer cells, PERK pathway activation might negatively regulate necroptosis. Conversely, Chen et al ^48^ observed that in hepatocytes from patients with chronic liver disease, ERS-induced PERK phosphorylation could lead to increased alpha-fetoprotein levels, resulting in necroptosis of hepatocytes. The regulatory outcome of the PERK pathway on necroptosis differs between cancerous and non-cancerous liver cells, especially when HBV infection occurs ^66^. In hepatocellular carcinoma, RIPK1/RIPK3/MLKL mediated necroptosis exists and is associated with immune infiltration and tumor development, and UPR/ERS is activated to influence tumor survival under tumor hypoxia and protein stress. Recently, studies have begun to unveil the regulatory role of the IRE1α signaling pathway in liver necroptosis. For example, in a study by Zhao et al ^67^ investigating the effects of *Yinchenhao Tang* on ameliorating the immune response suppression in fish induced by a high-carbohydrate diet, it was found that *Yinchenhao Tang* increased the expression of IRE1 and GRP78/BIP in hepatocytes, thereby alleviating hepatocyte necroptosis induced by the high-carbohydrate diet. As previously mentioned, excessive ERS can lead to cell death, suggesting that a moderated level of UPR may provide protective effects on liver cells.

xIn the pathogenesis of intestinal diseases such as inflammatory bowel disease (including ulcerative colitis and Crohn's disease) and necrotizing enterocolitis, the mutual promotion and vicious cycle between ERS and intestinal inflammation play a crucial role ^68^. In another study ^47^ using a dextran sulfate sodium-induced mouse model of inflammatory bowel disease, it was observed that a single cycle of dextran sulfate sodium did not significantly change PERK expression. However, levels of p-PERK, ATF6, IRE1α, RIPK1, and RIPK3 were all up-regulated, with the expression of RIPK1 and RIPK3 being higher in the distal colon than in the proximal colon. In mice treated with three cycles of dextran sulfate sodium, IRE1α and ATF6 were inhibited, but RIPK1/3 levels continued to increase. This indicates that in intestinal cells, ERS-induced necroptosis via UPR is evident, and ERS is more likely to induce necroptosis in the distal colon through UPR. Furthermore, during short-term ERS, all three UPR pathways may simultaneously regulate necroptosis, while prolonged ERS might lead cells to undergo necroptosis independent of the UPR pathways. In necrotizing enterocolitis, RIPK1/RIPK3/MLKL-dependent necroptosis is involved in the disease and can be inhibited, while TLR4-driven PERK–CHOP activation promotes cell death ^69^. ERS can directly drive intestinal epithelial necroptosis and cause colitis. Studies have confirmed that ERS induces necroptosis by inducing increased intestinal inflammatory responses in mice ^47^. The UPR transcription factor XBP1 interacts genetically with necroptotic colitis. IEC-specific deletion of XBP1 significantly exacerbates necroptotic colitis caused by CASP8 or FADD deficiency; conversely, RIPK3 or MLKL deficiency can block the occurrence of this colitis, suggesting a functional upstream-downstream association between the UPR's XBP1 axis and the RIPK3/MLKL axis ^70^. There are reports indicating that necroptosis plays an important role in intestinal diseases. In conditions like inflammatory bowel disease, colorectal cancer, and intestinal injury diseases, ERS can regulate necroptosis mediated by key kinases such as RIPK1 and RIPK3 by activating the UPR ^71^. In mouse and human intestinal ischemia-reperfusion models, UPR/ERS synergizes with RIPK3/MLKL-dependent necroptosis activation, and inhibiting apoptosis alleviates intestinal injury ^71^. Furthermore, numerous studies have confirmed that ERS and necroptosis regulate tumor cell progression in pancreatic cancer, gastric cancer, and other diseases. In pancreatic ductal adenocarcinoma, RIPK1/RIPK3/MLKL-mediated necroptosis correlates with disease behavior ^72^, while sustained UPR/ERS influences tumor adaptation and therapeutic responses ^73^. In gastric cancer, RIPK1/RIPK3/MLKL-dependent necroptosis-related molecules are associated with prognosis and tumor behavior ^74^^,^^75^, whereas UPR/ER stress activation contributes to tumor adaptation and progression in both primary tumors and precancerous lesions ^75^.

**Figure 4:**
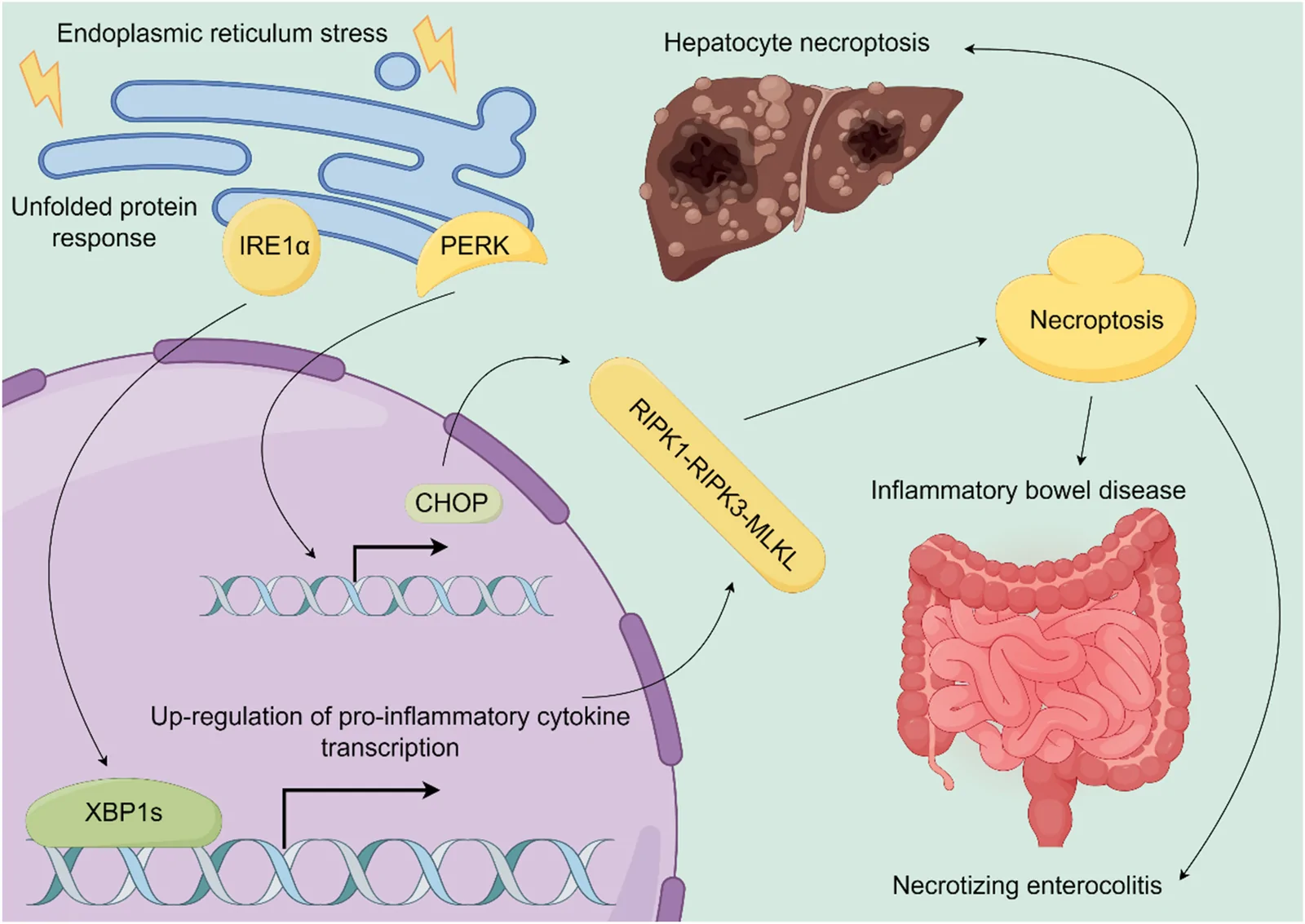
Endoplasmic reticulum stress can regulate necroptosis in digestive diseases through the downstream pathway PERK and IRE1α. The role of ATF6-mediated ERS in necroptosis during digestive disease pathogenesis remains unclear. The figure was created by Figdraw.

In summary, in the aforementioned digestive diseases, there is a complex interplay between ERS and necroptosis. They influence and promote each other, jointly participating in the pathophysiological processes of disease onset, development, and subsequent repair, which are crucial for intestinal health and disease outcomes (Fig. 4). However, the relationship between ERS and necroptosis in the digestive system still requires further in-depth research. Future studies should focus on the specific roles of these signaling pathways in digestive diseases and how they affect the survival and death of relevant cells, which is important for the development of new therapeutic strategies.

### Renal diseases

Necroptosis can lead to acute tubular necrosis, nephron loss, and maladaptive renal fibrosis. Research has shown that ERS can contribute to the formation of renal fibrosis by influencing the synthesis and degradation of the extracellular matrix ^76^. Additionally, necroptosis plays a role in the onset and progression of various kidney diseases, including acute kidney injury, chronic nephritis, and diabetic nephropathy ^77^^,^^78^. In diabetic nephropathy, specifically, ERS can regulate cell death through the three pathways not only in the kidney ^79^, but also inislet β-cells ^80^. Recent evidence indicates that during acute kidney injury, excessive activation of ERS can lead to apoptosis in renal cells ^76^. In chronic nephritis, the expression levels of ERS markers such as GRP78 and CHOP are significantly elevated, suggesting that ERS may promote apoptosis in glomerular and tubular cells by activating downstream apoptotic signaling pathways ^79^. Further research has confirmed that in renal cells, ERS can up-regulate inflammatory factors, thereby triggering the RIPK1/RIPK3/MLKL signaling pathway and resulting in necroptosis ^38^. In detail, up-regulation of GRP78 can activate the PERK pathway, leading to increased levels of inflammatory factors and triggering the RIPK1/RIPK3/MLKL signaling pathway, thereby causing necroptosis ^38^. This study also found that activation of the PERK pathway results in decreased ATPase activity and ATP content, as well as up-regulation of inflammatory cytokines like TNF-α, IL-1β, IL-6, and INF-γ, ultimately triggering RIPK1/RIPK3/MLKL-mediated necroptosis ^38^. These findings highlight the PERK pathway's role in regulating necroptosis in both renal and cardiac vascular smooth muscle cells by modulating inflammatory factors. In the renal ischemia-reperfusion and cisplatin-induced AKI models, RIPK1/RIPK3/MLKL-mediated necroptosis was clearly activated; inhibition or removal of this axis could reduce renal injury ^81^^,^^82^. In studies of non-kidney systems but with universal mechanisms, ER-stress can induce typical RIPK1/RIPK3/MLKL-dependent necroptosis through inflammatory factors IL-1β, IL-6, and TNF-α ^83^. The PERK/IRE1 signaling pathway in the UPR can up-regulate necroptosis-related signals. In CKD fibrosis models such as ureteral obstruction and adenosine diet, activation of the RIPK3/RIPK1/MLKL axis correlates with increased inflammation and matrix deposition. Targeting RIPK3 can reduce fibrosis, particularly through its MLKL-independent fibrotic effects mediated via the AKT–ACL pathway ^84^. In the oxalate nephropathy model, RIPK1/RIPK3/MLKL-dependent necroptosis was clearly activated ^85^. Genetic or pharmacological blockade of this axis can significantly reduce renal injury and crystal deposition ^86^. Meanwhile, high oxalate loading induces UPR/ERS responses in renal tissue and tubular epithelium ^87^.

**Figure 5:**
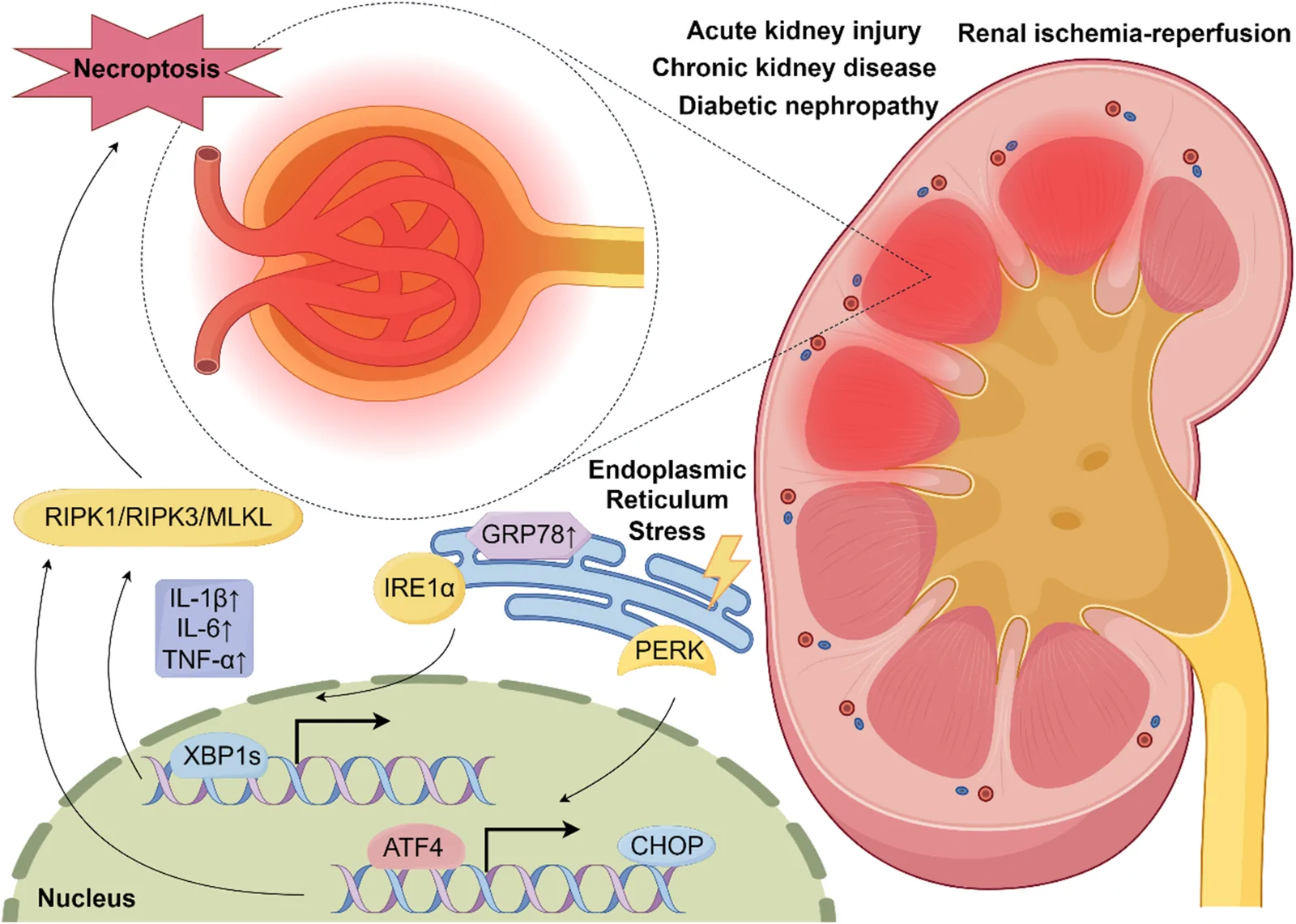
Endoplasmic reticulum stress can regulate necroptosis in renal diseases through the downstream pathway PERK and IRE1α by targeting RIPK1/RIPK3/MLKL signaling. The role of ATF6-mediated ERS in necroptosis during renal disease pathogenesis remains unclear. The figure was created by Figdraw.

Thus, ERS can regulate cell death in kidney-related diseases through both apoptosis and necroptosis mechanisms (Fig. 5). However, the specific role of this mechanism in other kidney diseases still requires further scientific investigation. It is also unclear whether cells in the same disease simultaneously undergo both forms of death and which mechanism is predominant. Therefore, in-depth research into the role of ERS in regulating necroptosis in kidney diseases is of significant scientific importance for developing new therapeutic strategies.

### Tumor

ERS and its downstream UPR pathways may also be present in tumors. At early dates, the potential function of this phenomenon was revealed in prostate cancer cells ^88^. In glioblastoma cells, a drug known as the carbonic anhydrase IX inhibitor S4 can trigger ERS while simultaneously activating the PERK and IRE1α pathways ^89^. Currently, research on necroptosis in the field of oncology is highly active, with literature confirming its crucial role in the treatment, prevention, and immune response of tumor cells ^90^^,^^91^. Previously, studies have demonstrated that ERS can regulate necroptosis in tumor cells. For instance, a standardized green tea extract, polyphenon E, can induce ERS and sustained UPR activation in prostate epithelial PC3 cells, leading to necroptosis ^88^. Recent publications have echoed similar findings. Reticulocalbin 1 (RCN1), an ER-resident Ca^2+^-binding protein, is dysregulated in cancer and present in prostate cancer cells. Depletion of RCN1 can lead to PERK activation and eIF2α phosphorylation, resulting in the suppression of prostate cancer cell proliferation and promoting cell death primarily through necroptosis ^92^. In prostate cancer, RIPK1/RIPK3/MLKL-mediated necroptosis can be induced and correlates with tumor behavior/treatment sensitivity ^92^. UPR, particularly IRE1α–XBP1s, remains active during tumor progression. In lung cancer, UPR/ERS is persistently activated and influences tumor adaptation and immune microenvironment interactions. RIPK1/RIPK3/MLKL-mediated necroptosis in NSCLC correlates with treatment efficacy or prognosis ^93^. In melanoma, RIPK1/RIPK3/MLKL-dependent necroptosis can be induced and relates to tumor behavior/immunointervention ^94^, while UPR/ERS signals, particularly IRE1α/ATF6, remain active and participate in tumor adaptation and drug resistance ^95^. In lymphoma, RIPK1/RIPK3/MLKL-mediated necroptosis is induced and depends on MLKL/RIPK3 ^96^. UPR/ERS remains active across different subtypes and affects tumor phenotype. In breast cancer, RIPK1/RIPK3/MLKL-mediated necroptosis is tumor biology-related, with upstream UPR/ERS signals being widely activated and pharmacologically modifiable ^97^. In head and neck squamous cell carcinoma, RIPK1/RIPK3/MLKL-mediated necroptosis can be induced and correlates with phenotype, while UPR/ERS signals remain continuously activated and influence tumor biology ^98^. In colorectal cancer, RIPK1/RIPK3/MLKL-mediated necroptosis can be induced and demonstrates tumor-suppressive potential, while UPR/ER stress is activated during tumor progression and treatment ^99^^,^^100^. In renal cell carcinoma, RIPK1/RIPK3/MLKL-mediated necroptosis has been demonstrated to exist and correlate with tumor phenotype ^101^.

These findings underscore the important role of ERS in regulating necroptosis in tumor cells. However, research on this mechanism in other tumor-related diseases is still lacking. If future research can develop inhibitors targeting these related signaling pathway proteins, it may lead to significant breakthroughs in cancer treatment.

## Conclusion and perspectives

**Figure 6:**
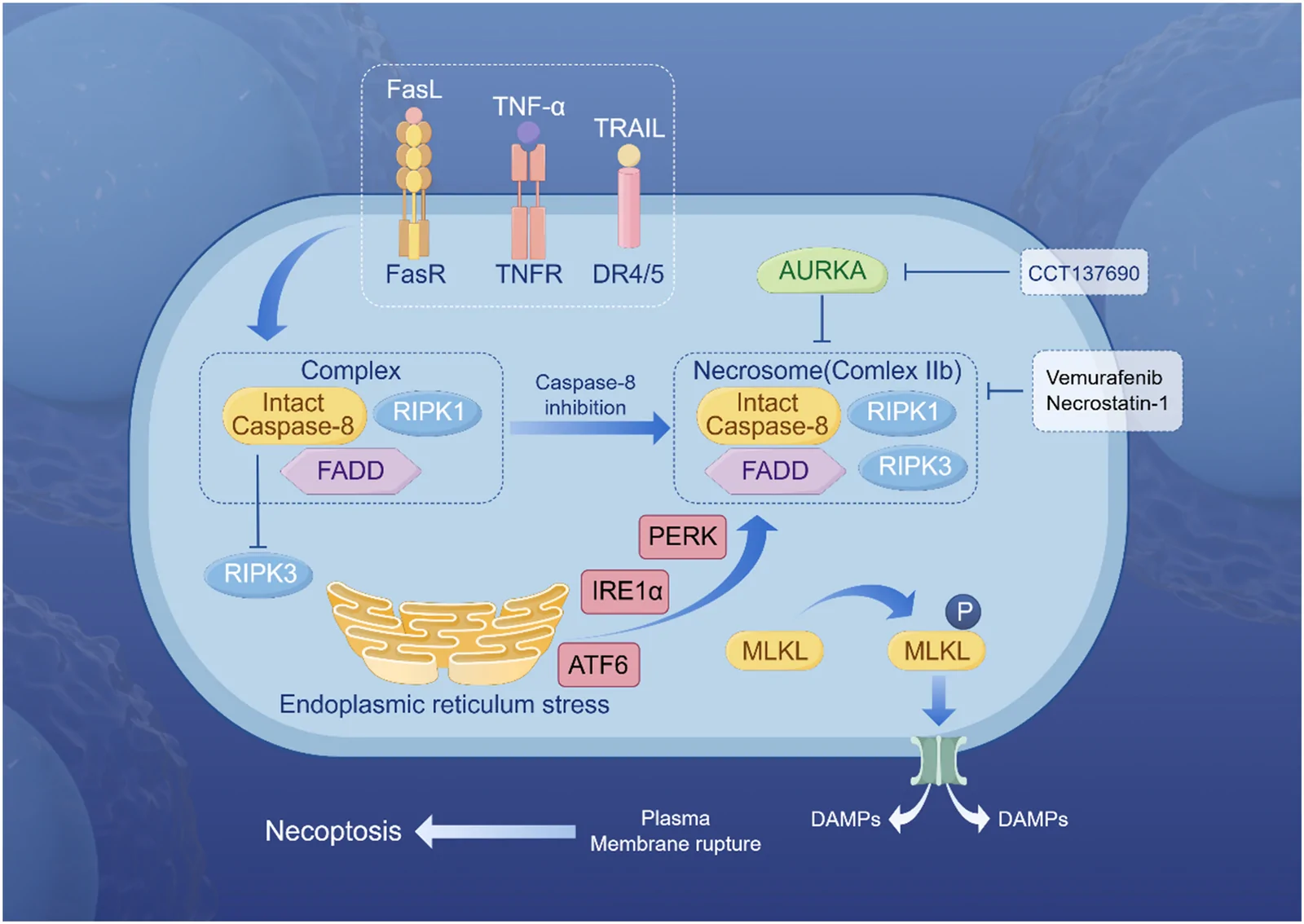
Endoplasmic reticulum stress can regulate necroptosis through the downstream activation of the PERK, IRE1α, and ATF6 pathways within the unfolded protein response. The figure was created by Figdraw.

In summary, current research has confirmed that ERS can regulate necroptosis through the downstream activation of the PERK, IRE1α, and ATF6 pathways within the UPR. Precisely, these downstream UPR pathways influence the activation of necrosome molecules including CASP8, RIPK1 and RIPK3, which may be inhibited by AURKA ^102^, necrostain-1, and vemurafenib ^103^ (Fig. 6). However, studies on the roles of the IRE1α and ATF6 pathways in regulating necroptosis are not yet comprehensive, and further expansion of research in this area is warranted. Mechanistically, how these three pathways specifically regulate RIPK1/RIPK3/MLKL-mediated necroptosis remains to be explored. It is still unclear whether the process involves the activation of RIPK1 and RIPK3 or directly activates MLKL. Moreover, whether ERS influences non-classical pathways of necroptosis is another intriguing avenue for exploration. Future studies should delve into the specific regulatory mechanisms of the UPR pathways in ERS on necroptosis and their roles across various systems. ERS leads to UPR, ER overload response, and CASP12-mediated apoptotic pathways. While the CASP12-mediated apoptotic pathway is not linked to necroptosis, the potential association between the ER overload response and necroptosis remains an enigma.

ERS-regulated necroptosis is implicated in numerous diseases, such as digestive, cardiovascular, urinary, and oncological diseases. The occurrence of necroptosis has been validated in various diseases within these systems, such as vascular smooth muscle cells in pulmonary hypertension, infarcted myocardial cells, renal epithelial cells in renal calcification, osteoblasts in osteoporosis, and prostate cancer cells. However, research in these areas is not yet comprehensive, indicating a need to expand studies on ERS-regulated necroptosis in the respiratory system, hematological disorders, reproductive system diseases, metabolic diseases, and infectious diseases. This will help confirm the actual presence and specific roles of related pathways and proteins in humans and animals.

Research into ERS modulators that may influence necroptosis is another significant future direction, and we listed them in Table 1. And some state-of-the-art techniques, including intracellular delivery ^104^ and extracellular vesicles ^105^, may provide more approaches for ERS modulator application. Known ERS modulators include TAK-243 (GRP78 inhibitors) ^106^, the peptide PYRAE (ERS trigger) ^107^, and BIP inducer X (ERS inhibitor) ^108^, which can modulate toxicity in certain cells or directly mediate apoptosis in them. The IRE1α pathway inhibitor KIRA6 is a small molecule that reduces cell death by inhibiting the activation of the IRE1α pathway ^109^. The PERK pathway inhibitor GSK2606414 not only inhibits the PERK pathway ^26^ to alleviate ERS but also plays a role in immunomodulation and enhancing anti-tumor immunity ^110^. ATF6 pathway inhibitors include Ceapins ^111^ and melatonin^112^, among which Ceapins selectively inhibit ATF6α without affecting ATF6β ^111^. ERS inhibitors might potentially affect multiple cell death pathways, including necroptosis. For example, one study showed that the deacetylated MDH1 and IDH1 can mitigate pan-apoptosis in hepatocytes, which includes pyroptosis, apoptosis, and necroptosis by inhibiting ERS ^113^. Furthermore, numerous natural compounds demonstrate the ability to modulate ERS and hold therapeutic potential for diseases associated with necroptosis. For example, glycyrrhizic acid alleviates myocardial ischemia-reperfusion injury in rats by attenuating ERS-induced apoptosis and inflammation, suggesting its potential as a therapeutic intervention ^114^. Another study demonstrates that phenylbutyrate treatment effectively reduces both ERS-related PERK/eIF2α pathway and pro-inflammatory cytokines, suggesting its therapeutic potential for Crohn's disease ^115^. Likewise, quercetin effectively ameliorates hyperuricemia-induced chronic kidney disease in rats by comprehensively modulating ERS-mediated inflammatory, fibrotic, and apoptotic pathways, showing superior renoprotective effects compared with febuxostat ^116^. MicroRNA has also been used for ERS regulation. For instance, miR-381-3p is an oncogenic miRNA that promotes renal cell carcinoma progression and confers a poor prognosis by suppressing both apoptotic and necroptotic cell death pathways, highlighting its potential as a therapeutic target ^117^. On the other hand, the impacts of necroptosis modulators such as AURKA inhibitor CCT137690^102^ on UPR/ERS remain to be further investigated.

**Table 1:** The modulators of unfolded protein response (UPR) in endoplasmic reticulum stress (ERS) that may influence necroptosis.

Modulators	UPR/ERS molecules	Necroptosis molecules	Diseases	Reference
BiP inducer X	BIP↑ GRP94↑ calnexin↑ CHOP↓	Cleaved CASP3↓	Chinese hamster ovary cells	^108^
Ceapins	ATF6α↓ GRP78↓ HERPUD1↓	/	U2-OS, HeLa, and HEK293T cells	^111^
Deacetylated MDH1 at K118 and IDH1 at K93	BIP↑ ATF6↑ XBP1↑ CHOP↑	RIPK1↑ GSDMD↑ CASP3↑ MLKL↑ IL-18↑ IL-1β↑	Acute liver failure	^113^
Forsythiaside A	GRP78↓ PERK ↓ elF2α↓ CHOP↓	IL-1β↓ IL-6↓ TNF-α↓	Acute kidney injury	^83^
Glycyrrhizic acid	GRP78↓ p-PERK↓ CHOP↓	TNF-α↓ IL-6↓	Myocardial ischemia–reperfusion injury	^114^
GSK2606414	PERK↓ NF-κB↓	MAPK↓	Osteoclast differentiation	26
GSK2656157	PERK↓	IFNγ↑	B16-F10 melanoma	^110^
KIRA6	IRE1α↓ NF-κB↓	CXCL8↓	A375, HeLa, HCT-116 and CT26 cells	^109^
Melatonin	ATF6↓	COX-2↓ Bcl-2↓ Bax↑	Hepatoma HepG2 cell	^112^
miR-381-3p	RIPK3↓ MLKL↓	TNF-α↓	Renal cell carcinoma	^117^
Pentapeptide PYRAE	ATF4↑ CHOP↑ cleaved CASP3↑ cl-PARP↑ LC3B↑	Apoptosis/necrosis↑	Breast cancer	^107^
Phenylbutyrate	EIF2AK3↓ DDIT3, STC2↓ DNAJC3↓ CALR↓ HSPA5↓ HSP90B1↓	IL-1β↓ IL-6↓ IL-17↓ TNF-α↓ sCD40L↓	Crohn's disease	^115^
Quercetin	GRP78↓ CHOP↓ p-PERK↓ IRE1α↓ ATF6↓	TLR4↓ NF-κB↓ IL-1β↓ TNF-α↓	Hyperuricemia	^116^
TAK-243	GRP78↑ p-PERK↑ p-EIF2α↑ ATF4↑ CHOP↑ p-IRE1α↑ Xbp1s↑	Apoptosis/necrosis↑ Bax ↑ Bcl-2 ↑	Glioblastoma	^106^

Research on ERS and its regulatory agents not only deepens our understanding of the cellular stress response mechanisms but also offers potential targets for developing new therapies for various diseases. Future research will continue to explore the interactions between ERS and other cellular signaling pathways and how precise regulation of ERS can promote health or treat diseases. Therefore, future research must delve deeply into the molecular mechanisms linking ERS and necroptosis and further investigate their potential connections with other modes of cell death. Additionally, the research scope should be broadened to explore their roles and effects in more systemic diseases. Our goal is to innovate new therapeutic approaches, unlock the vast potential for drug development, and ultimately translate these foundational research findings into clinical practice to benefit patients. With persistent effort and innovation, we have every reason to believe that research in this field will continue to unveil the mysteries of life and contribute significantly to the advancement of human health.

## CRediT authorship contribution statement

**Tong Li:** Writing – original draft, Investigation, Conceptualization. **Tian Su:** Writing – original draft, Investigation. **Sha Tang:** Resources, Investigation. **Kun Cao:** Investigation. **Pengbing Hua:** Investigation. **Jingbiao Gao:** Validation. **Hongwang Cui:** Writing – review & editing, Validation, Supervision, Funding acquisition, Conceptualization. **Tongmeng Jiang:** Writing – review & editing, Validation, Supervision, Funding acquisition, Conceptualization.

## Funding

This study was supported by the National Natural Science Foundation of China (No. 82502944, 81760260), Hainan Provincial Natural Science Foundation of China (No. 824QN270, 822RC827), and the Scientific Research Startup Foundation for Scholars of Hainan Medical University (China) (No. XRC2022019).

## Conflict of interests

The authors declared no competing interests.
